# A first-principles quantitative framework for how cohesin regulators shape chromatin loop extrusion

**DOI:** 10.1016/j.xgen.2026.101187

**Published:** 2026-03-11

**Authors:** Zibin Huang, Xinyi Liu, Junjun Ding

**Affiliations:** 1 Center for Stem Cell Biology and Tissue Engineering, Key Laboratory for Stem Cells and Tissue Engineering, Ministry of Education, Department of Histology and Embryology, Zhongshan School of Medicine, Sun Yat-sen University, Guangzhou, Guangdong, China

## Abstract

In this issue of *Cell Genomics*, Tortora and Fudenberg develop a first-principles framework in which loop extrusion is quantitatively regulated by multiple cohesin-associated factors, giving rise to “bursty extrusion.” This model predicts regulator-dependent changes in motor kinetics, chromatin contact patterns, and chromosome-scale morphology across spatial scales, providing a mechanistically grounded basis for quantitative modeling of 3D genome architecture.

## Main text

The three-dimensional organization of chromatin (3D genome) plays a crucial role in development and is closely linked to disease,^1^ reflecting its broad involvement in fundamental cellular processes such as gene expression, DNA damage repair, and stress responses. Chromatin folding spans multiple hierarchical scales—from local chromatin loops to large-scale chromosome morphology. A central mechanistic framework for understanding chromatin folding is loop extrusion, first proposed nearly a decade ago as a mechanistic model of 3D genome organization.^2^ In this model, loop-extruding factors such as cohesin translocate along DNA to progressively enlarge chromatin loops until they encounter barriers such as CTCF, the CCCTC-binding factor, giving rise to chromatin domains. Since then, a wealth of experimental evidence—from 3D genome data following perturbations of loop-extruding factors^3^ to single-molecule assays^4^—has supported loop extrusion as a core mechanism of interphase genome folding. Collectively, loop extrusion is now understood as a dynamic process through which cells actively shape their genomes.

Classical loop extrusion models typically treat the loop-extruding factor cohesin as a motor with uniform properties whose activity is modulated primarily by CTCF barriers.^2^ However, biochemical and cell biological studies have shown that cohesin functions together with multiple regulatory proteins—including the cohesin loading factor Nipped-B-like protein (NIPBL), the cohesin release factor wings apart-like protein (WAPL), and the cohesin-associated protein precocious dissociation of sisters protein 5 (PDS5)—whose perturbation profoundly alters cohesin dynamics and genome folding.^5^ A deeper mechanistic understanding of chromatin folding therefore requires dissecting the specific roles of these cohesin regulators. Still, this remains challenging because these factors interact with cohesin only transiently, with residence times of about one minute, and individual regulators often perform multiple functions.^5^ This highly dynamic behavior and functional complexity make it difficult to explain how short-lived interactions generate large and persistent changes in chromatin architecture. Previous models have not quantitatively captured the actions of these regulators and instead relied on fitting parameters to high-throughput chromosome conformation capture (Hi-C) data.^6^ Consequently, a mechanistically grounded framework linking the cellular abundance of cohesin and its regulators to loop extrusion kinetics and genome folding remains lacking.

**Figure 1:**
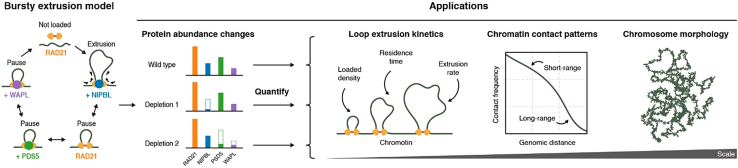
The bursty extrusion model links cohesin regulator abundance to genome organization across scales Tortora and Fudenberg derive a minimal chemical-reaction framework of loop extrusion (left) in which cohesin is modeled as a multi-state motor whose extrusion activity is regulated by NIPBL, PDS5, and WAPL. A central application of the model is its ability to predict how changes in cohesin regulator abundance influence loop extrusion kinetics (middle). When coupled to polymer simulations, the framework further predicts corresponding changes in chromatin contact patterns and chromosome morphology (right). Figure adapted from Tortora and Fudenberg.^5^

Tortora and Fudenberg fill this gap by formulating a chemical-reaction network that treats cohesin not as a single homogeneous motor but as a multi-state machine whose activity is modulated by the transient binding of distinct regulators. In their five-state network, NIPBL acts as the primary loader that enables productive loop extrusion, PDS5 facilitates WAPL recruitment and competes with NIPBL for cohesin binding, and WAPL serves as the principal unloader (Figure 1). As regulators bind to and dissociate from loaded cohesin on short timescales (∼1 min),^5^ cohesin cycles through distinct biochemical states, extruding only when associated with NIPBL and pausing when bound by other factors; this intermittent behavior is termed “bursty extrusion” (Figure 1). A key strength of the model is that its parameters are constrained by experimentally measured protein abundance, chromatin-bound fractions, and *in vivo* residence times, rather than fitted to Hi-C data. The model quantitatively captures regulator-specific perturbation phenotypes: NIPBL depletion reduces chromatin-bound cohesin, whereas WAPL or PDS5 depletion prolongs cohesin residence time and increases chromatin association to different extents, in agreement with measured bound fractions and residence times derived from the fluorescence recovery after photobleaching (FRAP) experiment.^7^ Collectively, this work establishes the first mechanistically grounded, quantitative framework that directly links the molecular kinetics of cohesin regulators to 3D genome architecture.

A key application of this bursty extrusion model is its ability to predict how changes in regulator abundance influence loop extrusion dynamics, including cohesin-loaded density, residence time, and extrusion rate (Figure 1). Altering protein abundance *in silico* reshapes the distribution of cohesin across biochemical states and thereby its extrusion behavior. On this basis, the authors find that extrusion dynamics depend nonlinearly on regulator abundance: changes in NIPBL, PDS5, or WAPL levels do not yield proportional shifts in motor properties, but instead generate distinct, regulator-specific dose-response behaviors. For example, varying levels of WAPL depletion markedly increase cohesin residence time and chromatin-bound fraction, whereas depletion of PDS5 produces different and non-monotonic effects on extrusion-related parameters, implying competition and multi-state transitions within the reaction network. These predictions agree with FRAP-derived residence times and experimentally measured chromatin-bound fractions under corresponding perturbations. Overall, this model demonstrates a nonlinear dependence of extrusion dynamics on regulator abundance within the reaction network.

This bursty extrusion framework further links regulator-dependent motor kinetics to genome-wide chromatin contact patterns (Figure 1). By coupling the reaction network to polymer simulations, the authors generated *in silico* Hi-C maps without fitting parameters to Hi-C data. Notably, the simulations reproduced the experimentally observed contact-versus-distance scaling curves, capturing the overall pattern of chromatin interactions from short- to long-range genomic distances and their shifts upon regulator depletion. In particular, altering WAPL or PDS5 levels shifted the characteristic “shoulder” of these curves toward longer genomic distances, indicating an increase in the typical genomic span of cohesin-mediated loops. In contrast, depletion of core cohesin components reduced long-range contacts, again consistent with experimental observations. Together, these results show that the model quantitatively predicts how molecular regulation of cohesin translates into global chromatin contact frequency patterns, bridging molecular kinetics with population-averaged genome architecture.

At larger scales, the bursty extrusion model further predicts how cohesin regulatory factors influence overall chromosome organization (Figure 1). Polymer simulations show that increasing cohesin residence time promotes the emergence of axial, “vermicelli-like” chromosome structures, consistent with experimental observations following WAPL depletion.^8^ The model provides a quantitative description of this transition and suggests that it arises from the increased accumulation of stably bound cohesin along the chromatin fiber, leading to axial compaction. In this framework, regulator-dependent changes in extrusion kinetics reshape not only local contact probabilities but also higher-order chromosome structure. Thus, the model links molecular binding dynamics to large-scale reorganization of entire chromosomes.

Looking ahead, this work lays the foundation for increasingly realistic and quantitative models of loop extrusion. Building on this framework, additional regulatory factors, post-translational modifications, and emerging structural insights into cohesin complexes could be incorporated to refine state transitions and motor behavior. Furthermore, integrating DNA sequence features and chromatin context may allow extrusion dynamics to vary along the genome, while cell-type-specific measurements of regulator abundance could enable predictive modeling across developmental stages. Recent evidence that cohesin dynamics are remodeled during cell fate transitions^9^ highlights the importance of extending such quantitative frameworks to diverse biological contexts, including development and disease.

More broadly, this work reflects a shift in the 3D genome field from identifying individual regulators toward understanding how multiple factors quantitatively cooperate to shape chromatin folding. While earlier efforts defined the key molecular players, the emerging challenge is to determine how their abundances, binding kinetics, and interactions collectively give rise to the physical genome structures observed in cells. In parallel, accumulating evidence suggests that loop extrusion operates broadly across species, and recent studies have inferred extrusion properties from cohesin distributions even in organisms lacking canonical CTCF barriers.^10^ Combining such approaches with quantitative reaction-based modeling could extend this framework beyond mammalian systems. The bursty extrusion model introduced here therefore marks an important step toward a predictive and mechanistically grounded theory of chromosome organization, linking first-principles biochemistry with polymer physics to explain how regulatory factors shape genome folding.

## Acknowledgments

Funding was provided by National Key Research and Development Program of China (2023YFA1800900) (J.D.), National Key Research and Development Program of China (2024YFA1106900) (J.D.), National Science Foundation for Distinguished Young Scholars of China (32425022) (J.D.), National Natural Science Foundation of China (32170798 and 32430031) (J.D.), Guangdong Innovative and Entrepreneurial Research Team Program (2016ZT06S029) (J.D.), National Natural Science Foundation of China (32400659) (X.L.), and China Postdoctoral Science Foundation (2023M744082) (X.L.).

## Declaration of interests

The authors declare no competing interests.
